# The two‐faced nature of BK polyomavirus: lytic infection or non‐lytic large‐T‐positive carcinoma‡

**DOI:** 10.1002/path.5127

**Published:** 2018-07-31

**Authors:** Volker Nickeleit, Harsharan K Singh, Daniel J Kenan, Piotr A Mieczkowski

**Affiliations:** ^1^ Division of Nephropathology, Department of Pathology and Laboratory Medicine The University of North Carolina Chapel Hill NC USA; ^2^ Department of Genetics The University of North Carolina Chapel Hill NC USA

**Keywords:** BK polyomavirus, immunosuppression, infection, large‐T, neoplasm, oncogenesis, polyomavirus nephropathy, transplantation, urogenital tract

## Abstract

In immunocompromised patients, reactivation of latent BK polyomavirus (BKPyV) can cause disease with lytic infections of the kidneys and the lower urinary tract. Emerging evidence also links BKPyV to oncogenesis and high‐grade intrarenal and transitional cell carcinomas. These neoplasms strongly express polyomavirus large‐T antigen as a defining feature; that is, they are ‘large‐T‐positive carcinomas’. Such neoplasms arise in immunocompromised patients, typically in renal allograft recipients, and preferentially in tissues harbouring latent BKPyV. In recent articles in this journal, it was shown that tumour cells harbour replication‐incompetent clonal BKPyV. The virus can be truncated and randomly integrated into the genome, and/or it can be mutated in an episomal state. Truncation and/or deletions in the BKPyV non‐coding control region can hamper late viral gene expression, replication, and cell lysis, while facilitating overexpression of early genes, including that encoding large‐T. Biologically active fusion proteins or alterations in human tumour suppressor or promoter function have not been described so far, making uncontrolled large‐T gene expression in non‐lytically infected cells a prime suspect for neoplastic transformation. Current concepts of BKPyV‐induced disease, including recent reports from this journal, are discussed, and evolving paradigms of BKPyV‐associated oncogenesis are highlighted. © 2018 The Authors. *The Journal of Pathology* published by John Wiley & Sons Ltd on behalf of Pathological Society of Great Britain and Ireland.

Polyomaviruses, including the BK polyomavirus (BKPyV) strain, are ubiquitous, and 40 years ago an editorial in the *Lancet* suggested that BKPyV was in search of a disease 1. The search has been successful, and there are two currently identified BKPyV‐driven diseases affecting the urogenital tract of immunocompromised patients: (1) productive/lytic infections with polyomavirus nephropathy (PVN) or haemorrhagic cystitis; and (2) non‐lytic polyomavirus large‐T‐expressing carcinomas.

Well studied are BKPyV infections that start as clinically insignificant primary events in young individuals. After primary infection, the virus is usually not completely cleared from the body, but rather establishes latency, mainly in the urogenital tract (renal epithelial cells and transitional cells), but also in other tissues and cells, such as tonsils, lymphocytes, blood mononuclear cells, and the brain. Latent BKPyV is usually in an episomal state and escapes standard morphological detection, necessitating the use of molecular techniques. Beyond infancy, latent infections can be found at any age, possibly driven by low‐level subclinical sporadic replication. By using polymerase chain reaction testing, we found latent BKPyV in the renal parenchyma (34%) and urothelium (42%) of asymptomatic adult patients (unpublished personal observations).

Under immune modulation, latent BKPyV can be reactivated and enter into a replicative cycle in permissive tissue. During the replicative cycle, early viral genes, including the large‐T gene, are expressed initially (easily detectable by immunohistochemistry; Table 1). Large‐T can stimulate its own expression by activating non‐coding control region (NCCR)‐regulated enhancers, sequestering p53, and allowing for phosphorylation of retinoblastoma proteins (Rbs), with subsequent activation of cell cycle proteins (immunohistochemical detection of Ki67; Table 1). Downregulation of early viral gene expression, including DNA synthesis and transcription of late BKPyV genes encoding capsid proteins, is controlled through cellular repressor proteins and microRNA feedback loops. These steps are required for the assembly of mature daughter virions, which are typically released from the host cell by cell lysis. Productive/lytic infections are often asymptomatic, transient events with viral activation limited to transitional cells lining the urothelium and so‐called decoy cell shedding in the urine, i.e. polyomavirus inclusion‐bearing cells 2. Marked lytic urothelial or renal BKPyV infection with associated inflammation characterises haemorrhagic cystitis or PVN. The incidence of haemorrhagic cystitis after bone marrow transplantation is 20% in adults and up to 40% in paediatric patients; PVN affects 6% of renal allograft recipients. By immunohistochemistry, host cells with replicating BKPyV express large‐T antigen, viral capsid proteins (VP1, VP2, and VP3), Ki67, and p53, whereas expression of p16 is largely absent (Table 1). Productive infections and associated tissue injury are reflected by high levels of BKPy viraemia, viruria or, in PVN, by urinary shedding of cast‐like viral aggregates, termed PV‐Haufen 3, 4. Once the immune system has recovered, productive infections are typically cleared via a cell‐mediated response 5, resulting in full structural recovery or, in protracted cases, such as PVN disease class III, in scarring 6. Productive/lytic infections, representing one category of BKPyV‐driven diseases, have four key elements: (1) tight regulation of the BKPyV replicative cycle through the NCCR; (2) transient and tightly regulated expression of the large‐T promoter; (3) episomal viral location; and (4) ultimate destruction of affected host cells carrying daughter virions.

**Table 1:** Immunohistochemical marker profile

BKPyV	Immunohistochemical marker profile	
Latent infection	BKPyV large‐T	Negative
	BKPyV VP1–VP3	Negative
	p16	Negative
	p53	Negative
	Ki67	Negative
Productive/lytic infection	BKPyV large‐T	Positive
	BKPyV VP1–VP3	Positive
	p16	Negative
	p53	Positive
	Ki67	Positive
Neoplastic cell transformation/large‐T‐positive carcinoma	BKPyV large‐T	Positive
	BKPyV VP1–VP3	Negative or only partially/incompletely expressed
	p16	Positive
	p53	Positive
	Ki67	Positive

BKPyV large T, BK‐virus large T protein expression; BKPyV VP, BK‐polyomavirus capsid protein (1, 2, 3) expression.

More recently, BKPyV has been associated with carcinogenesis along the urogenital tract in immunosuppressed renal allograft recipients. The neoplasms typically arise in the kidney transplant or the recipient's bladder 7, 8, 9. They show, as a defining feature, strong and diffuse expression of the large‐T promoter in neoplastic cell nuclei, i.e. large‐T‐positive carcinomas. However, viral replication is characteristically absent in the neoplastic tissue 10, although a concurrent productive/lytic infection might occasionally be found in adjacent non‐tumourous tissue compartments 11, 12. As compared with productive infections, BKPyV‐associated carcinomas are rare, usually high grade, and often difficult to classify (transitional cell, renal cell, or collecting duct), pointing towards currently poorly defined specific pathogenetic pathways. In one series, 20% of all genitourinary and renal carcinomas detected after kidney transplantation expressed large‐T antigen; all large T‐positive tumours were of urothelial origin with a fatal outcome in 25% of cases 7. One anecdotal case of a high‐grade donor‐derived metastatic BKPyV‐associated urothelial carcinoma recently reported in this journal went into complete remission following graft nephrectomy and discontinuation of immunosuppression; additional chemotherapy was not administered 13. Apparently, this carcinoma was rejected by the host. Although reports on BKPyV‐associated tumours have accumulated over the last decade, it was the discovery of Merkel cell polyomavirus and its oncogenic potential in skin tumours 14, 15 that stimulated further interest in BKPyV and its role in neoplastic transformation: is the virus a bystander or a driving force?

Three seminal publications by Kenan *et al* 12, 16 and Müller *et al* 13 reporting in‐depth molecular and deep gene sequencing data on three urogenital tumours collectively provided compelling evidence of BKPyV's central role in the pathogenesis of high‐grade human carcinomas arising in the urogenital tract of kidney transplant recipients. Kenan *et al* described two novel BKPyV genotype‐1a strains (called Chapel Hill BKPyV 1 and 2; GenBank accession #KP984526 and KY487998) in two neoplasms (each strain restricted to one tumour). Chapel Hill BKPyV 1 and 2 were linearised, truncated at viral capsid protein‐encoding sites, and randomly integrated into the tumour genome at a single locus. No BKPyV was found in adjacent non‐neoplastic parenchyma. Viral truncation with deletions in late gene sequences not only rendered Chapel Hill BKPyV 1 and 2 replication incompetent, but presumably also disrupted negative large‐T‐controlling feedback loops, resulting in unregulated and persistent expression of early viral genes. Overexpression of large‐T was presumably further promoted by deletions in the NCCR domain of Chapel Hill BKPyV 1 and resulting alterations in NCCR regulatory functions (deletion of the Q‐block and the R‐block from the OPQRS archetypical NCCR architecture). No role in oncogenesis could be attributed to host gene integration or novel bioactive fusion proteins. Müller *et al* also described a linearised and truncated BKPyV randomly integrated at a single locus into the tumour genome of a micropapillary metastatic large‐T antigen expressing urothelial carcinoma of donor origin. Similarly to the observations of Kenan *et al*, truncation was noted in late viral gene sequences, rendering the integrated virus replication incompetent. What is especially intriguing is that Müller *et al* additionally detected episomal full‐length BKPyV that was not replicating because of a short, specific 17‐bp deletion in the NCCR P‐block impairing late but not early viral gene expression. As the same NCCR deletion was noted in the integrated viral gene sequence, the authors speculated that, during neoplastic transformation, mutation of the episomal BKPyV had occurred first, followed by truncation and chromosomal integration. Sole episomal mutated BKPyV without integration into the tumour genome was seen in one of our recently diagnosed high‐grade large‐T‐positive renal cell carcinomas. This tumour‐associated full‐length BKPyV showed deletions in the R‐block and S‐block and duplications of the O‐block, P‐block and Q‐block of the NCCR, promoting early and hampering late viral gene expression. In contrast to the report of Müller *et al*, however, no deletions were found in the NCCR P‐block (personal observations). These modern‐era data are in line with those from older studies using electrophoresis and Southern blot techniques 17.

All studies combined show a common scheme in BKPyV‐associated cancer (Figure 1; Table 1): (1) occurrence in immunocompromised patients, often years after kidney transplantation; (2) high‐grade urothelial or renal (allograft) carcinomas, representing tissues commonly harbouring latent virus; (3) intraneoplastic clonal, mutated BKPyV; (4) diffuse, strong expression of polyomavirus large‐T transcripts and protein in dysplastic and neoplastic cells; (5) lack of intraneoplastic BKPyV replication/a productive infection, i.e. no or only partial expression of BKPyV capsid proteins VP1, VP2, and VP3 and no tumour‐derived BKPy viraemia or viruria; and (6) diffuse expression of p16.

**Figure 1:**
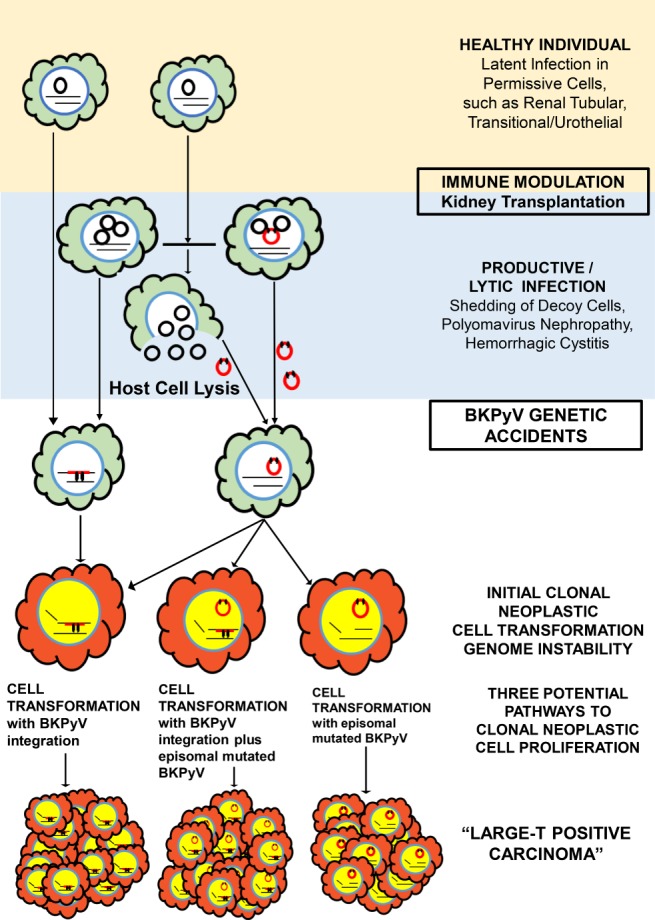
BKPyV in health and disease. In healthy individuals, BKPyV can remain in an episomal state and establish a latent infection in permissive cells, such as transitional and renal tubular cells. Immune modulation, including immunosuppression after kidney transplantation, promotes reactivation of latent BKPyV and lytic/productive infections. Asymptomatic subclinical latent infections or productive/lytic infections can show sporadic rare ‘genetic BKPyV accidents’ with mutated episomal and/or integrated viruses. Chromosomal integration is likely facilitated by mitotic activity and genomic instability. Mutated episomal BKPyV might be transmitted to permissive cells during lytic infections. Cells carrying mutated virus, regardless of whether episomal and/or integrated (three possible cell clones are illustrated in red), characteristically show high expression levels of early viral gene products. Late genes encoding capsid proteins and promoting cell lysis are not or only incompletely expressed, thereby hampering viral replication. As a consequence, cells with unchecked and prolonged overexpression of the early promoter large‐T have a ‘survival advantage’, show genomic instability, and can represent the nidus for clonal neoplastic proliferation. Illustrated at the bottom are three examples of clonal large‐T‐positive carcinomas driven by mutated episomal and/or integrated BKPyV. 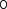, wild‐type episomal BKPyV; 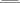, chromosomes; 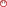, mutated replication‐incompetent episomal BKPyV (e.g. with rearrangements in the NCCR); 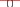, mutated BKPyV, truncated, linearised and randomly integrated at a single site into the host cell genome.

Current observations, based on experience with simian virus 40 (SV40) and Merkel cell polyomaviruses, have culminated in the following hypothetical model(s) of BKPyV‐associated carcinogenesis (Figure 1). In permissive cell types, impaired host immune surveillance allows for reactivation of latent BKPyV, productive infections, and viral replication, including rearrangements of the NCCR 18. In this microenvironment, sporadic severe ‘mutational accidents’ might occur, e.g. in the NCCR, that render episomal BKPyV replication incompetent while maintaining early viral gene expression. Such a scenario is suggested by Mueller *et al* 13 and was observed in one of our patients. Unchecked, persistent early BKPyV gene expression with large‐T promoter activity and impaired late gene expression inactivate p53 and Rb tumour suppressor functions, release E2F transcription factors, and promote cell entry into S‐phase. Virally induced host cell lysis is prevented. Crucial for cell transformation are not only the non‐lytic nature of the BKPyV infection but also intracellular large‐T concentrations high enough to effectively block p53 and phosphorylated Rb tumour suppressor functions 19. Cells in prolonged S‐phase, such as promoted by unchecked large‐T expression, show genomic instability with deletions, duplications, and translocations 20. Genomic instability probably also facilitates chromosomal integration of BKPyV. During these early events of oncogenesis, a neoplastic cell phenotype might be absent 20. As host cell lysis, as seen in productive infections, does not occur, cells carrying mutated BKPyV have a survival advantage, can replicate in a clonal fashion, and form the nidus for tumour growth. In this model, specific gene mutation(s) of episomal BKPyV constitute an essential initial step towards neoplastic cell transformation, which would therefore not require chromosomal integration as an indispensable event. Alternatively, in immunosuppressed individuals, genomic instability in multiplying cells carrying (latent) episomal BKPyV or productive infections might allow for accidental viral truncation and integration into the human genome, subsequent overexpression of large‐T, and neoplastic transformation (Figure 1). Immunohistochemically, transformed neoplastic cells express early BKPyV gene products (mainly large‐T), Ki67, p53, and p16 (Table 1). Late BKPyV gene products (VP1, VP2, and VP3) are conspicuously absent, or they might be dysfunctional and incompletely expressed 12, 21. In any event, suppressed immune surveillance provides a window of opportunity for cells containing aberrant virus to escape cytotoxic elimination, to transform, and to become ‘immortalised’. Cellular transformation and immortalisation are secondary results of non‐lytic infections with unchecked overexpression of large‐T. Whether large‐T‐positive carcinomas require additional chromosomal mutations for tumourigenesis is currently undetermined.

Recently collected evidence on the role of BKPyV as a neoplastic driver is compelling. Seminal observations made by Müller *et al* 13 and Kenan *et al* 12, 16 highlight biological/genetic BKPyV accidents that provide the right window of opportunity for neoplastic transformation. These pathways are similar to those described for SV40 and Merkel cell polyomaviruses. Future studies will undoubtedly further define paradigms, characterise crucial oncogenic BKPyV mutations, and elucidate the significance of mutated episomal versus integrated virus. Are large‐T‐expressing neoplasms also seen in tissues not harbouring latent BKPyV outside the urogenital tract? Can those tumours be seen in immunocompetent individuals/non‐renal transplant recipients, and are other chromosomal aberrations/oncogenes required as co‐stimulators to facilitate tumour growth? As latent JC polyomavirus (JCPyV) infections are also common in the urogenital tract, what is the oncogenic potential of JCPyV?

## Author contributions statement

All authors contributed to writing the manuscript.
